# Vascular Schizophrenia-like Psychosis in Older Adults

**DOI:** 10.3390/jcm12144831

**Published:** 2023-07-22

**Authors:** Michele Lauriola, Grazia D’Onofrio, Filomena Ciccone, Annamaria la Torre, Valentina Angelillis, Carmela Germano, Leandro Cascavilla, Antonio Greco

**Affiliations:** 1 Complex Unit of Geriatrics, Department of Medical Sciences, Fondazione IRCCS Casa Sollievo della Sofferenza, San Giovanni Rotondo, 71013 Foggia, Italy; m.lauriola@operapadrepio.it (M.L.); v.angelillis@operapadrepio.it (V.A.); c.germano@operapadrepio.it (C.G.); l.cascavilla@operapadrepio.it (L.C.); a.greco@operapadrepio.it (A.G.); 2 Clinical Psychology Service, Health Department, Fondazione IRCCS Casa Sollievo della Sofferenza, San Giovanni Rotondo, 71013 Foggia, Italy; f.ciccone@operapadrepio.it; 3 Laboratory of Gerontology and Geriatrics, Fondazione IRCCS Casa Sollievo della Sofferenza, San Giovanni Rotondo, 71013 Foggia, Italy; a.latorre@operapadrepio.it

**Keywords:** late-onset schizophrenia-like psychosis, vascular damage, cognitive impairment, biochemical concentrations, apolipoprotein E

## Abstract

Background: The aims of this study were to analyze prevalence and severity of vascular risk factors in older patients referred to our clinic due to onset of Very Late-Onset Schizophrenia-Like Psychosis (VLOSLP) and to create a specific phenotype based on pathophysiological insight rather than age of onset. Methods: In a longitudinal study, 103 (M = 39, F = 64; mean age of 80.32 ± 7.65 years) patients were evaluated with cognitive, neuropsychiatric, and functional assessment scales. Blood concentration of hemoglobin (Hb), mean corpuscular volume (MCV), platelets, total protein test (TPT), creatinine, azotemia, glycemia, total cholesterol (TC), triglycerides (TG), uric acid (UA), sodium (Na), potassium (K), chlorine (Cl), calcium (Ca), folate, vitamin B12 (Vit-B12), and homocysteine were measured. Presence/absence of tobacco use, alcohol consumption, psychoactive substance use, hypertension, hyperlipidemia, diabetes mellitus, and history of vascular disease were collected. Results: Females were more apathetic than males (NPI-Apathy: *p* = 0.040). Males had a significantly higher level of Hb (*p* = 0.019) and UA (*p* = 0.001), and a lower level of platelets (*p* = 0.004) and Ca (*p* = 0.003), and used more tobacco (*p* = 0.046) and alcohol (*p* = 0.024) than females. Comparing patients < 80 and ≥80 years, we found differences in frequency of vascular risk factors among men (*p* = 0.027). In total, 102 patients were treated for psychosis (59.16% of them were using atypical antipsychotics). Conclusions: The results of this study could be useful for a progressive demonstration of the causal relationship between cardiac and cerebral vascular events and VLOSLP.

## 1. Introduction

Schizophrenia (SZ) is a severe psychopathological syndrome characterized by delusions, hallucinations, and impaired skills and social relationships [1,2]. Usually, it first manifests in adolescence or early adulthood [3] but 20% of patients have their first onset after the age of 40 [4] and 1% of the older population present psychotic symptoms [5]. This is a frequent complication of cognitive impairment due to Alzheimer’s Disease or Vascular Dementia, especially in the more advanced stages of the diseases [6]. Otherwise, onset in older adults with normal cognitive functions is a rare condition but with a consequent worsening of functional autonomy and life expectancy [7]. For many years, there has been a growing interest in the relationship between behavioral disorders, aging, and vascular risk factors [8,9]. Since the first studies, these factors were linked to the presence of White Matter Hyperintensity (WMH) signals [10,11] and Brain Atrophy (BA) [12,13]. In particular, the early researchers began to investigate WMH and BA in subjects with schizophrenia [14,15] and showed that subjects with Late-Onset Schizophrenia have a greater prevalence rate of WMH [16]. This condition has been proven to be closely related to atherosclerosis [17], thromboembolism [18], and chronic cerebral hypoperfusion [19]. Indeed, Cardiovascular Disease (CVD) is much more prevalent in SZ than in the general population, and recent studies on functional genomics indicate that schizophrenia may be an adult vascular–ischemic disorder [20,21,22]. Endothelial Dysfunction (ED) has been described as a risk factor and mechanism in itself of the development of CVD [23,24]. In particular, the chronic inflammatory response [25] and the increased vasoconstriction [26] and oxidative stress [27] have attracted great interest in the relationship between CVD and SZ [28]. Regarding the diagnostic classification of patients, in relation to this problem, Geriatric Psychiatrists have published a review of the Conference of the International Late-Onset Schizophrenia Group. The authors concluded that Late-Onset Schizophrenia (LOS) begins after the age of 40 and bears a reasonable resemblance to early-onset schizophrenia (EOS), whereas Very Late-Onset Schizophrenia-Like Psychosis (VLOSLP) begins after the age of 60 and was best classified as having a schizophrenia-like psychosis on the basis of a convergence of clinical, epidemiological, neuroimaging, and neuropsychological data, although there was no consensus on the age limits for this distinction [29]. However, regarding the phenotypic characterization and clinical validity of LOP and VLOSLP, the discussion remains ongoing [30]. Indeed, some critical differences exist in their clinical presentation as compared with EOS. The results of various studies reported more positive symptoms in VLOSLP, a female preponderance, lower genetic risk, and higher rates of more-severe paranoid symptoms and persecutory delusions [31,32,33,34,35]. DSM-5 states that “late-onset cases can meet the diagnostic criteria for schizophrenia but it is not yet clear whether this is the same condition as schizophrenia diagnosed prior to mid-life” [36]. Discussion is needed on more than simply the cut-off for age of onset and clinical features. To date, it has not been clearly established whether it is a neurodegenerative pathology or a consequence of vascular damage. Investigating the neurobiology of VLOSLP is therefore the topic of our study. The aims of our study are

- to analyze the prevalence and severity of vascular risk factors in a population of older patients referred to our clinic due to the onset of VLOSLP;
- to create a specific phenotype based on pathophysiological insight rather than age of onset.

## 2. Materials and Methods

### 2.1. Study Sample

From January 2015 to December 2022, we screened older subjects who had consecutively attended the Cognitive Impairment Evaluation Unit of the Complex Unit of Geriatrics of Istituto di Ricovero e Cura a Carattere Scientifico (IRCCS) “Casa Sollievo della Sofferenza” for possible study enrollment. We obtained written informed consent for research from each patient, or from relatives or a legal guardian. 

All subjects were Caucasian, not including people of Jewish, Eastern European, or North African descent, with most individuals having Southern Italian ancestry, living in Southern Italy for at least three generations.

Inclusion criteria were (1) age ≥ 65 years; (2) diagnosis of Late-Onset Schizophrenia and Very-Late-Onset Schizophrenia-Like Psychosis according to International Late-Onset Schizophrenia Group (LOSG) criteria [29]; (3) absence of dementia; (4) presence of white matter hyperintensity based on computed tomography (CT scan); (5) treatment with acetylsalicylic acid already in progress; (6) ability to provide informed consent or availability of a relative or a legal guardian in the case of patients with severe cognitive impairment. Exclusion criteria were (1) presence of serious comorbidity, tumors, other diseases, or physiological status (ascertained blood infections, disorders of the thyroid, kidneys, or liver); (2) head trauma.

### 2.2. Study Design

The present study was conducted according to the Declaration of Helsinki, the Guidelines for Good Clinical Practice and the guidelines for Strengthening the Reporting of Observational Studies in Epidemiology, and it was approved by the local ethics committee for human experimentation (Prot. N. 3877/DS). This study was a longitudinal study, in which the assignment of an intervention to the participants, its effect assessment, and health-related biomedical or behavioral outcomes were not considered.

### 2.3. Cognitive, Neuropsychiatric, and Functional Assessment

In all patients, cognitive status was defined with the Mini-Mental State Examination (MMSE) [37], after a brief interview with the caregiver. 

The neuropsychiatric symptoms were assessed using the Neuropsychiatric Inventory (NPI) [38], which consisted of 12 neuropsychiatric domains: delusions, hallucinations, depression, anxiety, agitation/aggression, euphoria, disinhibition, irritability/lability, apathy, aberrant motor activity, sleep disturbance, and eating disorder. 

In all patients, functional status was assessed by activities of daily living (ADL) [39] and instrumental activities of daily living (IADL) [40] scales.

### 2.4. Genotyping

Genotyping was conducted on blood samples. Apolipoprotein E (ApoE) alleles (corresponding to allele combinations at single nucleotide polymorphism (SNP) +3937/rs429358 and SNP + 4075/rs7412) were genotyped using the ABI 7900 Taqman (Applied Biosystems, Foster City, CA, USA) system. The two ApoE single nucleotide polymorphisms exist at amino acids 112 and 158, which were targeted by the Taqman probes. The individual genotypes at the two sites were then combined to create a single standard ApoE genotype.

### 2.5. Value Quantification of Biochemical Concentrations

The blood samples (3–5 mL of blood) were collected intravenously from an upper limb of each patient in the morning. Then, blood samples were stored in Vacutainer tubes containing citrate; within not more than 30 min, the samples were transferred to the department of biochemistry and analyzed in a full autoanalyzer. 

The blood concentration of hemoglobin (Hb), mean corpuscular volume (MCV), platelets, total protein test (TPT), creatinine, azotemia, glycemia, total cholesterol (TC), triglycerides (TG), uric acid (UA), sodium (Na), potassium (K), chlorine (Cl), calcium (Ca), folate, vitamin B12 (Vit-B12), and homocysteine were measured in all patients.

### 2.6. Vascular Risk Factor Assessment

Through a semi-structured interview, medical history and milestones from the patient’s life were assessed as shown below: (1) lifetime tobacco use, (2) alcohol consumption, (3) psychoactive substance use and abuse, (4) history of vascular disease (hypertension, hyperlipidemia, diabetes mellitus, stroke, myocardial infarction, atrial fibrillation, and heart failure), and (5) anti-smooth muscle antibody (ASMA). 

Following the guidelines for the diagnosis and management of hypertension in adults, hypertension was defined as systolic blood pressure > 140 mmHg, diastolic blood pressure > 90 mmHg, or as currently receiving antihypertensive treatment [41]. 

Hyperlipidemia was defined according to the guidelines for management of dyslipidemia and prevention of cardiovascular disease [42]. All recruited patients with hyperlipidemia were in treatment with statins.

Diabetes mellitus was defined according to the Consensus Statement by the American Association of Clinical Endocrinologists and American College of Endocrinology on the Comprehensive Type 2 Diabetes Management Algorithm [43]. 

At the time of the medical visit, all patients were in treatment with acetylsalicylic acid; the patients with atrial fibrillation were in oral anticoagulant therapy.

All patients had performed a neuroimaging examination (CT scan) in order to highlight the presence of white matter hyperintensity. The focal cortical–subcortical outcomes were reported in patients with previous stroke.

### 2.7. Statistical Analyses

For dichotomous variables, hypotheses regarding differences between the groups were tested using chi-square test. This analysis was made using the 2-Way Contingency Table Analysis. For continuous variables, normal distribution was verified by the Shapiro–Wilk normality test and the one-sample Kolmogorov–Smirnov test. For normally distributed variables, hypotheses regarding differences among the groups were compared by means of Welch’s two-sample *t*-test or analysis of variance (ANOVA) under a general linear model. For non-normally distributed variables, hypotheses regarding differences among the groups were compared by means of the Wilcoxon rank sum test with continuity correction or by means of the Kruskal–Wallis rank sum test. Risks (adjusted by cognitive impairment presence/absence and severity) are reported as odds ratios (OR) along with their 95% confidence interval (CI). All the statistical analyses were made with the R Ver. 2.8.1 statistical software package [The R Project for Statistical Computing; available at URL http://www.r-project.org/ (accessed on 14 February 2023)]. Tests in which the *p* value was smaller than the type I error rate α = 0.05 were declared significant.

## 3. Results

During the enrollment period, 181 older patients were screened for inclusion in the study. Of these, 12 patients were excluded because they were younger than 60 years, 58 had a diagnosis of dementia, 6 had not run a CT scan, and 2 had an incomplete examination. Thus, the final population included 103 patients, comprising 39 men (37.86%) and 64 women (62.14%) with a mean age of 80.32 ± 7.65 years and a range from 65 to 95 years. 

### 3.1. Demographic, Cognitive, Functional, Neuropsychiatric, and Clinical Characteristics 

As shown in Table 1, dividing patients by gender, there was only a significant difference in NPI-Apathy: females were more apathetic than males (*p* = 0.040).

As shown in Table 2, dividing patients by gender, males had a significantly higher level of Hb (*p* = 0.019) and UA (*p* = 0.001), and a lower level of platelets (*p* = 0.004) and Ca (*p* = 0.003), than females; in terms of vascular risks, males used more tobacco (*p* = 0.046) and alcohol (*p* = 0.024) than females.

### 3.2. Vascular Risk Frequency 

As the data in Table 3 show, we analyzed separately the frequency of vascular risk factors in patients < 80 years compared to patients ≥ 80 years, looking for differences according to age in the distribution of risk factors. We only found differences in the frequency of vascular risk factors comparing these two age strata for frequency of MI among men (*p* = 0.027).

### 3.3. Current Use of Antipsychotic and Antidepressant Drugs

Regarding psychiatric and clinical treatment (Table 4), 102 patients were treated for psychosis (75 with affective psychosis and 27 with no-affective psychosis), and 59.16% of them were using atypical antipsychotics (AA). The most commonly used drugs were serotonin selective reuptake inhibitor (SSRI) drugs (26 (26.52%) patients, 5 with no-affective psychosis) and typical antipsychotic (TA) drugs (17 (17.51%) patients, 8 with no-affective psychosis).

## 4. Discussion

In this study, we explored the neuropsychiatric profile of older people with psychotic symptoms arising in a state of normal cognitive function, paying close attention to vascular risk factors. We studied our patients, first of all, divided by gender and noting that most are female as in previous studies [44,45,46]. In our results, there are not only more females, but there is a greater degree of severity of psycho-behavioral symptoms as noted in the total NPI score that is higher than in males. Particularly in females, hallucinations, anxiety, and depressive symptoms are more severe. Males, on the other hand, express symptoms more frequently characterized by irritability and aggressiveness. To characterize the symptoms and investigate their onset and course, for each patient, the interview with the family was fundamental. All patients were described as having normal cognitive functions and complete independence in activities of daily living until the onset of psychiatric symptoms, after which they came to the attention of our clinic. Screening of cognitive functions, performed with the MMSE, shows a score below normal. This data must be considered in relation to the poor collaboration of all patients and the opposition shown towards the neuropsychometric tests that we attempted to perform. Finally, there were no differences between males and females regarding laboratory analyses performed to analyze blood count, glycolipid profile, liver and kidney function, folic acid, and vitamin B12. 

### 4.1. Homocisteine and ApoE Gene 

We analyzed homocysteine levels and the presence of the ɜ4 allele of the ApoE gene [47,48] considered as a link between vascular risk factors and brain damage. Homocysteine showed a higher concentration in the male population and the ɜ4 allele of the ApoE gene was more present in the female population. To the best of our knowledge, our work provides more information about the neuropsychiatric profile of onset of psychotic symptoms at an older age than other studies [34,35,49,50,51,52,53,54,55,56].

### 4.2. Endothelial Dysfunction

In addition, we focused on the prevalence of vascular risk factors and previous acute cardiac and cerebral vascular events. We also considered previous Acute Myocardial Infarctions (AMI) as an expression of systemic ED [53], which many studies associate with schizophrenia [57,58,59]. As mentioned, after analyzing the neuropsychiatric profile of our patients and dividing them into subgroups considering gender differences and age of onset < 80 and ≥80, our interest turned to the study of the causes. To deepen our understanding, we started from the anamnesis, focusing in particular on vascular risk factors, which became the subject of this study. Vascular risk factors we mean chronic systemic diseases, which cause ED, increase the likelihood of cardiac and cerebral vasculopathy, and put the patient at increased risk of Acute Vascular Events (AVE) [60]. The main piece of data to report is the prevalence of previous AVE, which in our population is 27.2%. Specifically, 14.6% reported a previous cerebral Ischemic Stroke (IS) with functional recovery, and 12.6% reported a previous AMI. Analyzing data, by gender and age, we found that in the male population over 80 years of age, the prevalence of previous AVE is 47.6%. In particular, previous IS is 23.8% and previous AMI is 23.8%. In the female population aged over 80, on the other hand, the prevalence of AVE is 29.4%. Specifically, previous IS is 11.8% and previous AMI is 17.6%. Moreover, the most evident piece of data is the constant comorbidity present in our patients. The pathologies most frequently known many years before the onset of psychotic symptoms are arterial hypertension, atrial fibrillation, heart failure, type 2 diabetes mellitus, and dyslipidemia under treatment. With our results, it is possible to hypothesize the predominantly vascular genesis of schizophrenia with onset after 80 years. ED could be the neurobiological substrate that predisposes patients to the onset of psycho-behavioral disorders, as shown by studies for which its markers are associated with significant changes in regional Cerebral Blood Flow (rCBF) [61]. In this regard, recent evidence suggests that apathy, depression, fatigue, and delirium are associated with rCBF changes, in particular hypoperfusion in the bilateral temporal lobes [62]. 

### 4.3. Neurovascular Unit Dysfunction

Finally, regarding the role of ED in neurobiology of schizophrenia, we share the recent theory of Neurovascular Unit (NU) dysfunction [63]. NU consists of the brain’s microvessels, pericytes, glial cells (astroglia, microglia, oligodendroglia), and neurons. It is the structure that arises from the interaction between glia, neurons, and the cerebral microvascular endothelium [64,65,66]. There are many clinical and experimental data that have studied the link from neuroinflammation, oxidative stress, and genetic factors to clinical and pathological findings suggestive of NU dysfunction in schizophrenia [67,68,69,70,71,72,73,74]. 

### 4.4. Vascular Schizophrenia-like Psychosis as Specific Phenotype

**Figure 1:**
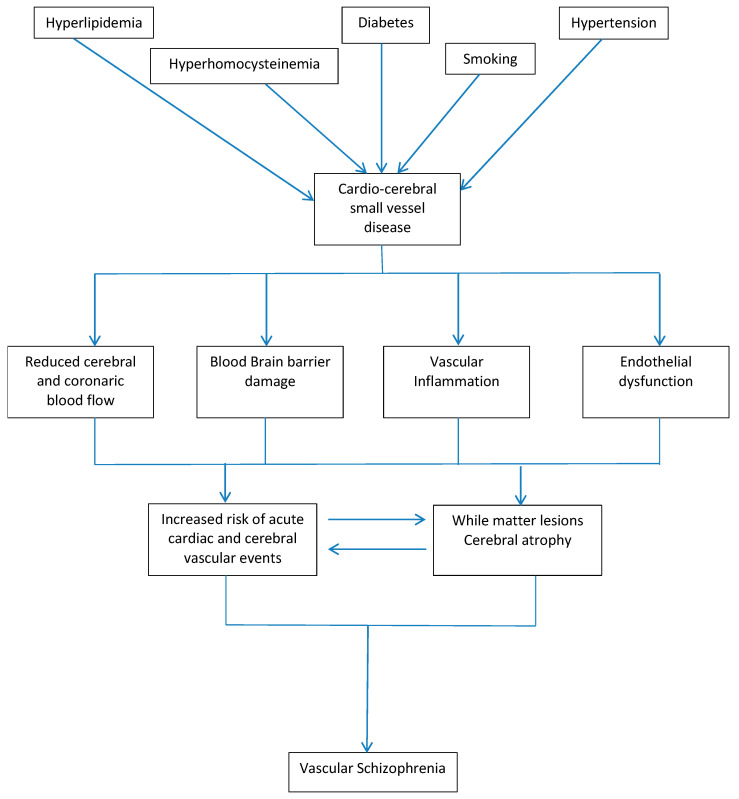
Flow chart of possible mechanisms of Vascular Schizophrenia-Like Psychosis.

We therefore deem it necessary, with regard to “vascular depression”, which is known as a specific subtype distinct from nonvascular forms [75], and with regard to psychosis, to have a particular phenotype from which a different consequent prevention, follow-up, and treatment can be developed [76]. A more specific association between cerebral vasculopathy and psychotic disorders has been formalized by Steffans and Krishnan, who proposed diagnostic criteria based on known stroke or transient acute vascular event, radiological findings of hyperintensity, cortical or subcortical infarcts, age of onset after 50 years, and reduction in cognitive, executive, and information-processing speed [77]. The efficacy, in these patients, of typical and atypical antipsychotics, antidepressants, and mood stabilizers [78], also tested by us, raises the interesting question, to be explored in future studies, of the mechanism by which vascular dysfunction affects neurotransmissions’ dopaminergic, serotonergic, and membrane activity. Our work adds some data in the context of uncertainty and interest where there is a lack of large longitudinal observational studies, which is certainly difficult due to the complexity of the patients. Our results could be useful for a progressive demonstration of the causal relationship between vascular pathology and VLOSLP and therefore the definitive validation of “Vascular Schizophrenia-Like Psychosis” (Figure 1) in research and clinical practice.

## Figures and Tables

**Table 1:** Demographic, cognitive, functional, and neuropsychiatric characteristics of patients according to gender.

	ALL N = 103	Male N = 39	Female N = 64	*p*-Value
**Age** (years)				0.639
Mean ± SD	80.32 ± 7.65	80.78 ± 8.08	80.04 ± 7.42	
Range	65.00–95.36	65.00–95.36	65.00–93.45	
**MMSE**				0.727
Mean ± SD	20.55 ± 5.98	20.81 ± 7.18	20.39 ± 5.17	
Range	0–30.00	0–30.00	0–30.00	
**ADL**				0.348
Mean ± SD	4.57 ± 1.58	4.38 ± 1.56	4.69 ± 1.59	
Range	1.00–6.00	2.00–6.00	1.00–6.00	
**IADL**				0.415
Mean ± SD	3.38 ± 2.92	3.08 ± 2.75	3.56 ± 3.02	
Range	0–8.00	0–8.00	0–8.00	
**NPI—Total score**				0.770
Mean ± SD	40.73 ± 22.39	39.90 ± 19.64	41.23 ± 24.04	
Range	4.00–100.00	6.00–84.00	4.00–100.00	
**NPI—Distress**				
Mean ± SD	15.78 ± 7.33	14.82 ± 7.18	16.36 ± 7.41	0.304
Range	0–38.00	0–32.00	4.00–38.00	
**NPI—Delusion**				
Mean ± SD	5.11 ± 4.74	5.00 ± 4.58	5.17 ± 4.86	0.859
Range	0–12.00	0–12.00	0–12.00	
**NPI—Hallucination**				
Mean ± SD	4.73 ± 4.85	3.56 ± 4.48	5.44 ± 4.97	0.057
Range	0–15.00	0–15.00	0–15.00	
**NPI—Agitation/Aggresion**				
Mean ± SD	5.57 ± 4.27	6.44 ± 4.43	5.05 ± 4.11	0.109
Range	0–16.00	0–16.00	0–12.00	
**NPI—Depression**				
Mean ± SD	5.40 ± 4.38	5.13 ± 4.67	5.56 ± 4.22	0.628
Range	0–16.00	0–16.00	0–12.00	
**NPI—Anxiety**				
Mean ± SD	3.93 ± 4.62	3.44 ± 4.76	4.23 ± 4.54	0.397
Range	0–16.00	0–16.00	0–12.00	
**NPI—Euphoria**				
Mean ± SD	0.88 ± 2.87	0.62 ± 2.38	1.05 ± 3.14	0.462
Range	0–12.00	0–12.00	0–12.00	
**NPI—Apathy**				
Mean ± SD	4.89 ± 4.75	3.67 ± 4.62	5.64 ± 4.71	**0.040**
Range	0–12.00	0–12.00	0–12.00	
**NPI—Disinhibition**				
Mean ± SD	0.79 ± 2.30	0.82 ± 2.32	0.77 ± 2.31	0.907
Range	0–9.00	0–9.00	0–9.00	
**NPI—Irritability**				
Mean ± SD	5.04 ± 4.62	5.62 ± 5.08	4.69 ± 4.31	0.325
Range	0–16.00	0–16.00	0–12.00	
**NPI—Abnormal activity**				
Mean ± SD	1.01 ± 2.70	0.87 ± 2.32	1.09 ± 2.93	0.688
Range	0–12.00	0–9.00	0–12.00	
**NPI—Sleep disturbance**				
Mean ± SD	4.97 ± 4.24	4.28 ± 3.95	5.39 ± 4.38	0.189
Range	0–12.00	0–9.00	0–12.00	
**NPI—Eating disorders**				
Mean ± SD	1.86 ± 3.62	1.03 ± 2.66	2.38 ± 4.03	0.066
Range	0–12.00	0–12.00	0–12.00	
**Psychosis**				
Affective—n (%)	76(73.8)	26(34.2)	50(65.8)	0.200
No-affective—n (%)	27(26.2)	13(48.1)	14(51.9)	

Legend: MMSE, Mini-Mental State Examination; ADL, Activities of Daily Living; IADL, Instrumental Activities of Daily Living; NPI, Neuropsychiatric Inventory.

**Table 2:** Clinical and lifestyle characteristics of patients according to gender.

	ALL N = 103	Male N = 39	Female N = 64	*p*-Value
*Laboratory measurements*				
**ApoE**				
ε2/ε3–N (%)	6 (5.8)	3 (7.7)	3 (4.7)	
ε3/ε3–N (%)	61 (59.2)	24 (61.5)	37 (57.8)	0.862
ε3/ε4–N (%)	27 (26.2)	9 (23.1)	18 (28.1)	
ε4/ε4–N (%)	9 (8.7)	3 (7.7)	6 (9.4)	
**Hb, g/dL**				**0.019**
Mean ± SD	13.40 ± 1.52	13.85 ± 1.62	13.13 ± 1.39	
Range	10.00–18.00	10.00–18.00	10.00–17.00	
**MCV, fL**				0.195
Mean ± SD	90.84 ± 6.55	91.91 ± 6.58	90.18 ± 6.50	
Range	59.00–100.00	65.00–100.00	59.00–100.00	
**Platelets, mil/mcl**				**0.004**
Mean ± SD	220.94 ± 79.94	192.15 ± 61.82	238.48 ± 84.94	
Range	21.00–595.00	21.00–367.00	119.00–595.00	
**TPT, g/d** **L**				0.768
Mean ± SD	6.99 ± 0.71	6.96 ± 0.63	7.01 ± 0.75	
Range	5.00–9.00	5.00–8.00	5.00–9.00	
**Creatinine, mg/dL**				0.406
Mean ± SD	1.01 ± 0.36	1.04 ± 0.33	0.98 ± 0.38	
Range	0–3.00	1.00–2.00	0–3.00	
**Azotemia, mg/dL**				
Mean ± SD	45.95 ± 16.81	45.64 ± 17.49	46.14 ± 16.53	0.884
Range	21.00–106.00	21.00–105.00	22.00–106.00	
**Glycemia, mg/dL**				
Mean ± SD	96.90 ± 24.56	95.15 ± 21.40	97.98 ± 26.44	0.574
Range	68.00–187.00	68.00–164.00	71.00–187.00	
**TC, mg/dL**				
Mean ± SD	183.46 ± 45.21	175.33 ± 46.09	188.41 ± 44.30	0.156
Range	97.00–301.00	106.00–301.00	97.00–298.00	
**TG, mg/dL**				
Mean ± SD	114.83 ± 85.66	130.79 ± 128.31	105.09 ± 41.20	0.140
Range	39.00–835.00	42.00–835.00	39.00–311.00	
**UA, mg/dL**				
Mean ± SD	4.90 ± 1.55	5.53 ± 1.52	4.53 ± 1.46	**0.001**
Range	2.00–10.00	2.00–10.00	2.00–9.00	
**Na, mmol/L**				
Mean ± SD	140.57 ± 3.60	139.95 ± 3.03	140.95 ± 3.88	0.171
Range	120.00–147.00	133.00–146.00	120.00–147.00	
**K, mmol/L**				
Mean ± SD	4.28 ± 0.63	4.25 ± 0.40	4.29 ± 0.74	0.780
Range	3.00–8.00	4.00–5.00	3.00–8.00	
**Cl, mmol/L**				
Mean ± SD	104.89 ± 3.67	104.82 ± 2.94	104.94 ± 4.08	0.876
Range	87.00–116.00	87.00–116.00	87.00–116.00	
**Ca, mg/dL**				
Mean ± SD	9.04 ± 0.59	8.82 ± 0.48	9.17 ± 0.61	**0.003**
Range	8.00–11.00	8.00–10.00	8.00–11.00	
**Folate, ng/mL**				
Mean ± SD	7.62 ± 6.56	7.20 ± 7.26	7.93 ± 6.06	0.629
Range	2.00–41.00	2.00–41.00	2.00–41.00	
**Vit-B12, pg/mL**				
Mean ± SD	371.04 ± 286.85	323.91 ± 205.32	405.60 ± 333.33	0.216
Range	60.00–2000.00	60.00–806.00	98.00–2000.00	
**Homocysteine, μmol/L**				
Mean ± SD	12.58 ± 5.34	13.97 ± 6.83	11.66 ± 3.90	0.100
Range	4.00–28.00	4.00–28.00	5.00–20.00	
*Vascular risks*				
**Hypertension**				
Yes—N (%)	57 (55.3)	20 (51.3)	37 (57.8)	0.518
No—N (%)	46 (44.7)	19 (48.7)	27 (42.2)	
**Hyperlipidemia in treatment**				
Yes—N (%)	22 (21.4)	7 (17.9)	15 (23.4)	0.510
No—N (%)	81 (78.6)	32 (82.1)	49 (76.6)	
**Diabetes**				
Yes—N (%)	20 (19.4)	6 (15.4)	14 (21.9)	0.419
No—N (%)	83 (80.6)	33 (84.6)	50 (78.1)	
**AF**				
Yes—N (%)	11 (10.7)	5 (12.8)	6 (9.4)	0.583
No—N (%)	92 (89.3)	34 (87.2)	58 (90.6)	
**HF**				
Yes—N (%)	8 (7.8)	4 (10.3)	4 (6.2)	0.461
No—N (%)	95 (92.2)	35 (89.7)	60 (93.8)	
**Stroke**				
Yes—N (%)	15 (14.6)	6 (15.4)	9 (14.1)	0.854
No—N (%)	88 (85.4)	33 (84.6)	55 (85.9)	
**MI**				
Yes—N (%)	13 (12.6)	5 (12.8)	8 (12.5)	0.962
No—N (%)	90 (87.4)	34 (87.2)	56 (87.5)	
**Tobacco use**				
Yes—N (%)	5 (4.9)	4 (10.3)	1 (1.6)	**0.046**
No—N (%)	98 (95.1)	35 (89.7)	63 (98.4)	
**Alcohol consumption**				
Yes—N (%)	3 (2.9)	3 (7.7)	0	**0.024**
No—N (%)	100 (97.1)	36 (92.3)	64 (100.0)	

Legend: ApoE, apolipoprotein E; Hb, hemoglobin; MCV, mean corpuscular volume; TPT, total protein test; TC, total cholesterol; TG, triglycerides; UA, uric acid; Na, sodium; K, potassium; Cl, chlorine; Ca, calcium; Vit-B12, vitamin B12; AF, atrial fibrillation; HF, heart failure; MI, myocardial infarction.

**Table 3:** Frequency of vascular risk factors in patients less than and more than 80 years old, per gender.

	<80 Years	≥80 Years	*p*-Value
*Males*			
**Hypertension**			
Yes—N (%)	8 (44.4)	12 (57.1)	0.429
No—N (%)	10 (55.6)	9 (42.9)	
**Hyperlipidemia in treatment**			
Yes—N (%)	4 (22.2)	3 (14.3)	0.520
No—N (%)	14 (77.8)	18 (85.7)	
**Diabetes**			
Yes—N (%)	4 (22.2)	2 (9.5)	0.273
No—N (%)	14 (77.8)	19 (90.5)	
**AF**			
Yes—N (%)	1 (5.6)	4 (19.0)	0.209
No—N (%)	17 (94.4)	17 (81.0)	
**HF**			
Yes—N (%)	2 (11.1)	2 (9.5)	0.871
No—N (%)	16 (88.9)	19 (90.5)	
**Stroke**			
Yes—N (%)	1 (5.6)	5 (23.8)	0.115
No—N (%)	17 (94.4)	16 (76.2)	
**MI**			
Yes—N (%)	0	5 (23.8)	**0.027**
No—N (%)	18 (100.0)	16 (76.2)	
**Tobacco use**			
Yes—N (%)	3 (16.7)	1 (4.8)	0.222
No—N (%)	15 (83.3)	20 (95.2)	
**Alcohol consumption**			
Yes—N (%)	1 (5.6)	2 (9.5)	0.643
No—N (%)	17 (94.4)	19 (90.5)	
*Females*			
**Hypertension**			
Yes—N (%)	16 (53.3)	21 (61.8)	0.496
No—N (%)	14 (46.7)	13 (38.2)	
**Dyslipidemia**			
Yes—N (%)	9 (30.0)	6 (17.6)	0.244
No—N (%)	21 (70.0)	28 (82.4)	
**Diabetes**			
Yes—N (%)	9 (30.0)	5 (14.7)	0.140
No—N (%)	21 (70.0)	29 (85.3)	
**AF**			
Yes—N (%)	1 (3.3)	5 (14.7)	0.119
No—N (%)	29 (96.7)	29 (85.3)	
**HF**			
Yes—N (%)	0	4 (11.8)	0.052
No—N (%)	30 (100.0)	30 (88.2)	
**Stroke**			
Yes—N (%)	5 (16.7)	4 (11.8)	0.573
No—N (%)	25 (83.3)	30 (88.2)	
**MI**			
Yes—N (%)	2 (6.7)	6 (17.6)	0.185
No—N (%)	28 (93.3)	28 (82.4)	
**Tobacco use**			
Yes—N (%)	1 (3.3)	0	0.283
No—N (%)	29 (96.7)	34 (100.0)	
**Alcohol consumption**			
Yes—N (%)	0	0	1.000
No—N (%)	30 (100.0)	34 (100.0)	

Legend: AF, atrial fibrillation; HF, heart failure; MI, myocardial infarction.

**Table 4:** Current use of antipsychotic and antidepressant drugs in our sample.

Medications	Affective Psychosis	No-Affective Psychosis	*p*-Value
**Untreated**—N (%)	1 (1.3)	0	0.549
**Treated**—N (%)	75 (98.7)	27 (100.0)	
**SSRI**—N (%)	21 (27.6)	5 (18.5)	0.349
**AA**—N (%)	41 (53.9)	17 (63.0)	0.417
**TA**—N (%)	9 (11.8)	8 (29.6)	**0.032**
**MS**—N (%)	14 (18.4)	1 (3.7)	0.063
**Tz**—N (%)	11 (14.5)	2 (7.4)	0.342

Legend: SSRI, selective serotonin reuptake inhibitors; AA, atypical antipsychotic; TA, typical antipsychotic; MS, mood stabilizers; Tz, trazodone.

## Data Availability

Not applicable.
